# Supplementary material for: Comparative Transcriptomic Analysis of Temozolomide Resistant Primary GBM Stem-Like Cells and Recurrent GBM Identifies Up-Regulation of the Carbonic Anhydrase CA2 Gene as Resistance Factor
Source: Cancers (Basel). 2019 Jun 30;11(7):921. doi: 10.3390/cancers11070921 (PMC6678269; doi:10.3390/cancers11070921)
Supplement: Supplementary file 1 [file cancers-11-00921-s001.pdf]

Supplementary Materials

Comparative Transcriptomic Analysis of Temozolomide Resistant Primary GBM Stem-Like Cells and Recurrent GBM Identifies Up-Regulation of the Carbonic Anhydrase *CA2* Gene as Resistance Factor

Ricarda Hannen, Martin Selmansberger, Maria Hauswald, Axel Pagenstecher, Andrea Nist, Thorsten Stiewe, Till Acker, Barbara Carl, Christopher Nimsky and Jörg Walter Bartsch

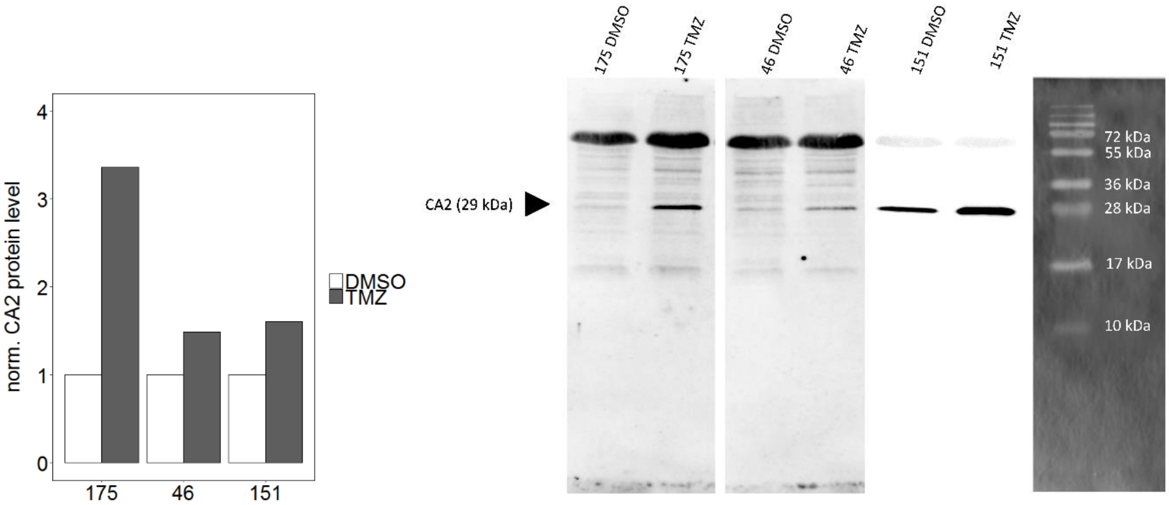

Figure S1. Supplementary Materials of Western Blot for Figure 5C.

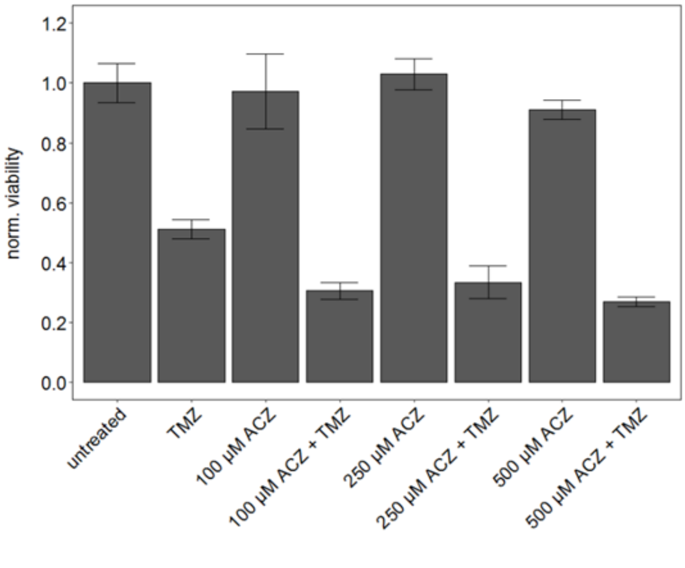

Figure S2. Higher ACZ concentrations were combined with the IC_{50} of TMZ (here 250 µM) in a pilot study of $n = 1$ using the TMZ resistant cells 175. Note, that using higher concentrations than 100 µM did not lead to a more pronounced effect.

© 2019 by the authors. Licensee MDPI, Basel, Switzerland. This article is an open access article distributed under the terms and conditions of the Creative Commons Attribution (CC BY) license (http://creativecommons.org/licenses/by/4.0/).
